# Cost-Effectiveness of Routine Third Trimester Ultrasound Screening for Fetal Growth Restriction Compared to Care as Usual in Low-Risk Pregnancies: A Pragmatic Nationwide Stepped-Wedge Cluster-Randomized Trial in The Netherlands (the IRIS Study)

**DOI:** 10.3390/ijerph19063312

**Published:** 2022-03-11

**Authors:** Jens Henrichs, Ank de Jonge, Myrte Westerneng, Viki Verfaille, Arie Franx, Henriëtte E. van der Horst, Judith E. Bosmans, on behalf of the IRIS Study Group

**Affiliations:** 1 Amsterdam University Medical Center, Vrije Universiteit Amsterdam, Department of Midwifery Science, AVAG and Amsterdam Public Health, Van der Boechorststraat 7, 1081 BT Amsterdam, The Netherlands; m.westerneng@amsterdamumc.nl (M.W.); verfaillevumc@gmail.com (V.V.); 2 Amsterdam University Medical Center, Vrije Universiteit Amsterdam, Department of Midwifery Science, AVAG and Amsterdam Reproduction and Development, Van der Boechorststraat 7, 1081 BT Amsterdam, The Netherlands; ank.dejonge@amsterdamumc.nl; 3 Department of Obstetrics and Gynecology, Erasmus Medical University Center, Doctor Molewaterplein 40, 3015 GD Rotterdam, The Netherlands; a.franx@erasmusmc.nl; 4 Amsterdam University Medical Center, Vrije Universiteit Amsterdam, Department of General Practice, Amsterdam Public Health, Van der Boechorststraat 7, 1081 BT Amsterdam, The Netherlands; he.vanderhorst@amsterdamumc.nl; 5 Department of Health Sciences, Faculty of Science, Vrije Universiteit Amsterdam, Amsterdam Public Health Research Institute, Van der Boechorststraat 7, 1081 BT Amsterdam, The Netherlands; j.e.bosmans@vu.nl

**Keywords:** routine third trimester ultrasonography, severe adverse perinatal outcome, cluster-randomized trial, economic evaluation

## Abstract

Routine third trimester ultrasonography is increasingly used to screen for fetal growth restriction. However, evidence regarding its cost-effectiveness is lacking. We aimed to evaluate the cost-effectiveness of routine third trimester ultrasonography to reduce adverse perinatal outcomes compared to usual care (selective ultrasonography). An economic evaluation alongside a stepped-wedge cluster-randomized trial was conducted. Via 60 midwifery practices 12,974 Dutch women aged ≥16 years with low-risk pregnancies were enrolled at 22.8 (SD = 2.4) weeks’ gestation. All practices provided usual care. At 3, 7, and 10 months a third of the practices were randomized to the intervention strategy providing routine ultrasonography at 28–30 and 34–36 weeks’ gestation and usual care. The primary clinical outcome was a dichotomous composite measure of 12 severe adverse perinatal outcomes (SAPO) up to one week postpartum. Information on perinatal care and societal costs was derived from Netherlands Perinatal Registry, hospital records and a survey. Cost-effectiveness analyses revealed no significant differences in SAPO and healthcare and societal costs between the intervention strategy (*n* = 7026) and usual care (*n* = 5948). Cost-effectiveness acceptability curves showed that the probability of cost-effectiveness was never higher than 0.6 for all possible ceiling ratios. Adding routine third trimester ultrasonography to usual care is not cost-effective in reducing SAPO.

## 1. Introduction

Monitoring fetal growth is a crucial objective of antenatal care [1]. Being small-for-gestational-age (SGA), i.e., fetal size or birth weight below the 10th percentile for a certain gestational age, is a risk factor for perinatal morbidity and mortality, and adverse long-term outcomes, such as cardiovascular disease in adulthood [2,3,4]. In practice, SGA is used as a proxy for fetal growth restriction (FGR). When SGA is detected, the fetus may be small but healthy or the fetus may be small as the result of FGR. A French population-based study (*n* = 480,448) demonstrated that the pre- and postnatal healthcare costs for a SGA neonate in 2011 were EUR 2783 higher than for an appropriate-for-gestational-age (AGA) neonate [5]. It was estimated that maternal and neonatal hospital costs related to SGA contributed 23% to the total costs for maternity care in France [5].

Timely detection and adequate clinical management of FGR during (late) pregnancy is expected to reduce adverse effects and costs, but is only possible when effective screening procedures and subsequent adequate clinical management are available. Currently, many Western countries, such as the Netherlands, selectively use ultrasonography, i.e., serial fundal height (SFH) measurements and ultrasonography if clinically indicated, to monitor fetal growth. Interestingly, a previous cohort study in the UK (*n* = 3977) showed that routine third trimester ultrasonography approximately tripled the detection rate (sensitivity = 57%) of SGA neonates compared to selective ultrasonography (sensitivity = 20%) [6]. Contrastingly, a meta-analysis (*n* = 34,980) including 13 randomized trials did not show positive effects of routine third trimester ultrasonography on primary outcomes, including perinatal mortality, preterm birth, and Caesarean section rates [7]. In line with these findings, a recent stepped-wedge cluster randomized trial in the Netherlands (*n* = 13,046) conducted by our research group also showed that routine third trimester ultrasonography had no effect on severe adverse perinatal outcomes [8].

Although routine third trimester ultrasound screening for FGR has not been proven to be clinically effective so far, [7,8] Dutch healthcare providers have increasingly proposed to offer routine third trimester ultrasonography for this purpose [9]. The idea of offering standard third trimester ultrasound scans is also popular among the majority of Dutch pregnant women [10]. However, the impact of implementing routine third trimester ultrasonography into antenatal care on health care costs is unclear. A recent decision tree analysis showed that routine third trimester ultrasonography examining fetal size was not cost-effective [11]. However, cost-effectiveness studies alongside controlled trials that directly compare routine and selective third trimester ultrasonography are lacking. Therefore, a pragmatic large-scale nationwide stepped-wedge cluster-randomized trial was conducted in the Netherlands comparing these two strategies among low-risk pregnancies [8]. The cluster randomization led to the roll-out of the intervention and prevented contamination bias related to preferences of women for or against third trimester ultrasound scans [8]. The stepped-wedge design made it possible for numerous midwifery practices to participate, even if these practices preferred one of the strategies. Based on this design, each midwifery practice first offered usual care and then changed to applying routine third trimester ultrasonography at a pre-defined moment in the course of the study in line with the randomization scheme. In the current study, we evaluated the cost-effectiveness of routine third trimester ultrasonography in combination with usual care to reduce severe adverse perinatal outcomes in comparison to usual care alone. Both strategies comprised a multidisciplinary protocol for the detection and management of FGR.

## 2. Materials and Methods

### 2.1. Study Design and Study Population

This economic evaluation was conducted alongside a nationwide stepped-wedge cluster-randomized trial, the IUGR Risk Selection (IRIS) Study [12]. In short, between 1 February 2015 and 29 February 2016, 60 primary care midwifery practices enrolled 13,520 pregnant women who had a low-risk singleton pregnancy at enrolment [8]. On average, women were enrolled at 22.8 weeks gestation (SD = 2.4) [8]. Pregnant women with a low risk status were included in the study if they received maternity care in one of the participating midwifery practices at enrolment, were aged at least 16 years, had a singleton pregnancy, no serious obstetric or medical risk factors, and a reliable expected date of birth derived from a dating scan or a reliable record of the first day of their last menstrual period [8].

All practices started with the control strategy offering usual care (serial fundal height measurements and clinically indicated third trimester ultrasonography only) [8]. At 3, 7, and 10 months, one third of the midwifery practices were randomized to the intervention strategy offering routine third trimester ultrasonography, i.e., one routine biometry scan at 28–30 weeks’ gestation and one at 34–36 weeks’ gestation in addition to usual care [8]. For both strategies in the trial, the same multidisciplinary protocol for the detection and management of FGR was used [13]. The design of the IRIS study has previously been described in more detail [12].

All participating women provided written informed consent. The IRIS study has been approved by The Dutch Institutional Review Board of the VU Medical University (reference number: 2013.409) and has been registered at the Netherlands Trial Register (NTR4367).

### 2.2. Data Collection

Costs and clinical data were collected using the following four sources: the database of the Netherlands Perinatal Registry (Perined), sonographic databases, hospital records of mothers and their neonates, and a longitudinal survey embedded in the IRIS study [12].

#### 2.2.1. Primary Clinical Perinatal Outcome

The primary clinical outcome was a composite measure of 12 severe perinatal outcomes based on information from the Perined database and hospital records [8]. This composite measure comprised one or more of the following: perinatal death between 28 weeks’ gestation and the first week after birth; five minute Apgar score < 4; impaired consciousness; asphyxia; seizures; >24 h assisted ventilation; septicemia; meningitis; bronchopulmonary dysplasia; intraventricular hemorrhage; cystic periventricular leukomalacia; and necrotizing enterocolitis [8,12].

#### 2.2.2. Pregnancy-Related Healthcare and Sonography Costs

Pregnancy-related healthcare costs were retrieved from the Perined database, covering information for the period from approximately 22 weeks’ gestation until one week postpartum [12]. Cost items from the Perined database comprised information on consultations with the obstetrician and/or pediatrician, medical transport (ambulance), duration of hospital admission (at the neonatal unit), obstetric interventions during and after labor, and type and place of birth (see Appendix B). Moreover, information was available on the number of third trimester ultrasound scans in primary care, extracted from the sonographic databases of the participating midwifery practices and sonography centers.

#### 2.2.3. Hospital Care Costs

Using digital standardized case report forms, five trained research assistants collected additional in-depth perinatal clinical data and maternal and neonatal cost data from hospital files [8,12]. Data from the hospital files of neonates (and their mothers) were retrieved if severe adverse perinatal outcomes were suspected based on the Perined database [8]. Suspected severe adverse perinatal outcomes included perinatal death, an Apgar score of <4 five minutes after birth, a birth weight below the 2.3rd centile (or a birth weight ranging from the 2.3rd to 5th centile and) and being admitted to neonatal care for more than three days or being referred to a neonatologist (if information on duration of admission was lacking or not clearly recorded in the Perined database) [8,12].

Moreover, in-depth cost data based on hospital records of mothers participating in the survey were collected in case of a maternal hospital admission during pregnancy or postpartum, a maternal hospital admission of more than 48 h after a birth without medical intervention or an admission of more than 72 h related to a caesarean section. To ensure an adequate retrieval of data from the hospital records by the research assistants, experts in neonatology, obstetrics and economic evaluation research helped to operationalize the respective measures.

Cost data for the period from 22 weeks’ gestation until 6 months postpartum were collected using hospital records. Cost items included medical transports (ambulance), laboratory tests or other diagnostic tests (ultrasound examinations, blood tests, X-ray, MRI, genetic tests), CTG monitoring, duration of (postpartum) hospital admissions, interventions during admission to the neonatal unit, examinations and interventions and treatment during the perinatal/postpartum period (see Appendix B for more detail).

#### 2.2.4. Societal Costs

Alongside the cluster-randomized trial, a longitudinal survey was conducted among a non-selective subsample of pregnant women participating in the IRIS study [12,14]. By participating midwifery practices or via e-mail, a non-selective subgroup of 2467 women was consecutively invited to participate in this survey between May and December 2015 [14]. Of these 2467 women, 1475 (59.8%) pregnant women provided additional informed consent for participation in the survey. These women were asked to fill in online questionnaires on societal costs at baseline (around 22 weeks’ gestation) and at 6 weeks and 6 months after the estimated date of birth. Using validated self-report instruments quality-of-life, healthcare utilization (not directly related to pre- and postpartum medical care), and productivity losses were assessed [12].

#### 2.2.5. Quality of Life

As part of the survey, maternal pre- and postpartum quality of life was measured using the EQ-5D-5L [15]. The EQ-5D-5L health states were converted to utilities using the Dutch EQ-5D-5L tariff [16]. Subsequently, QALYs were calculated using linear interpolation between the measurement time points. Higher QALY scores reflect better quality of life.

#### 2.2.6. Costs and Costing

Healthcare utilization and lost productivity were measured using adapted versions of the iMTA Medical Cost Questionnaire (iMCQ) and the iMTA Productivity Cost Questionnaire (iPCQ), respectively [17].

Healthcare costs were calculated based on standard costs published in Dutch costing guidelines or in previous economic evaluations of perinatal care in the Netherlands [18,19,20,21,22,23,24,25,26,27,28]. Medication costs were calculated using prices of the Netherlands National Healthcare Institute [21].

The friction cost approach (friction period 85 days) was used to estimate lost productivity costs using Dutch rates [18,29]. The friction cost approach assumes that a sick employee is replaced after a certain amount of time (the friction period) after which there are no lost productivity costs anymore [18,29].

Appendix B presents an overview of the cost categories included in the economic evaluation and prices used. All costs presented were adjusted to the year 2015 using consumer price indices if necessary. The year 2015 was chosen because most data were collected in that year.

### 2.3. Statistical Analysis

The economic evaluation related the difference in costs between pregnant women receiving routine third trimester ultrasonography and those receiving usual care to the differences in clinical effects. We performed both cost-effectiveness and cost-utility analyses, applying various perspectives and time horizons. The statistical analyses were conducted according to the intention-to-treat principle.

Firstly, we conducted a cost-effectiveness analysis for the composite outcome measure of severe adverse perinatal outcome from a healthcare perspective (analysis 1). The time horizon of the first analysis ranged from 22 weeks’ gestation to one week postpartum. For this first analysis, pregnancy-related data obtained from the Perined database of all women (*n* = 12,974) included in the current study population were used. Cost and effect differences were estimated using a bivariate regression model. Bias-corrected and accelerated bootstrapping with 5000 replications was performed to estimate statistical uncertainty.

**Figure 1:**
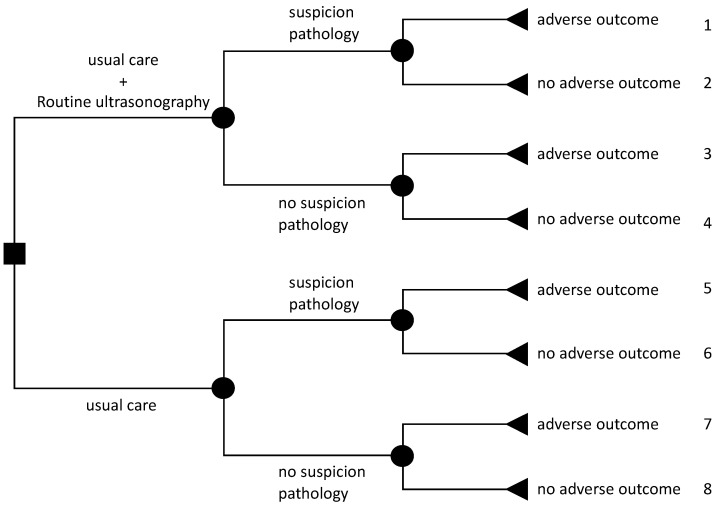
Decision tree showing possible outcome pathways.

Secondly, we conducted two cost-effectiveness analyses for the composite of severe adverse perinatal outcome from a societal perspective (analysis 2). The time horizon of these analyses ranged from 22 weeks’ gestation until, respectively, one week postpartum and 6 months postpartum. Pregnancy-related data obtained from the Perined database of all 12,974 women were also used for these analyses. In addition, detailed cost data from hospital records, and self-reported healthcare utilization and lost productivity data of women were extrapolated to estimate societal costs for the complete study population. To do this, a decision tree was developed that includes all possible outcomes for the two treatment conditions, leading to eight possible pathways and outcome strategies (Figure 1). For each pathway, mean pregnancy-related healthcare, hospital and other societal costs per woman were estimated. For each pathway, costs were weighted by the probability of occurrence based on the decision tree. To estimate uncertainty, a probabilistic sensitivity analysis was performed. For each of the cost categories, a gamma distribution was fitted. Using Monte Carlo simulation techniques, 1000 random draws were taken from these distributions. Uncertainty around cost and effect differences was estimated using 95% credibility intervals (CrI) by estimating the 2.5% and 97.5% percentiles.

Finally, we conducted a cost-utility analysis from a societal perspective with a time horizon ranging from 22 weeks’ gestation to 6 months after the estimated date of birth (analysis 3). For this cost-utility analysis, we used available data of the women in the non-selective survey sample. Missing cost and effect data were imputed by applying multiple imputation using the MICE algorithm by Van Buuren et al., (1999) [30]. Predictive mean matching was used in the imputation procedure to rectify the skewed distribution of costs. The number of imputed datasets was increased until the loss of information was less than 5% (m = 5) [31]. Cost and effect differences were estimated using a bivariate regression model. Bias-corrected and accelerated bootstrapping with 5000 replications was performed to estimate statistical uncertainty.

Incremental Cost-Effectiveness Ratios (ICER) were computed by dividing the difference between the groups in healthcare costs by difference in effects. For severe adverse perinatal outcomes, the difference between groups was multiplied by −1 to ensure that positive differences indicate that routine third trimester ultrasound screening is more effective than usual care and negative differences that routine third trimester ultrasound screening is less effective than usual care. Uncertainty surrounding the ICERs was graphically illustrated for cost-effectiveness planes for all conducted analyses. Cost-effectiveness acceptability curves were estimated to illustrate the probability that routine third trimester ultrasonography to screen for FGR is cost-effective in comparison to usual care for a range of different ceiling ratios, thereby showing decision uncertainty.

## 3. Results

For 12,974 women (96.0% women of the included 13,520 women; 7026 intervention and 5948 usual care), information on pregnancy-related healthcare costs could be retrieved from the Perined database and data on the severe adverse perinatal composite outcome were available. Of the 2339 maternal and/or neonatal cases (1308 intervention and 1031 usual care) included in an additional in-depth data-collection based on hospital records, cost data were available for 1515 cases (755 intervention and 760 usual care). Of the 1475 respondents (952 intervention and 523 usual care) enrolled for the non-selective survey, societal costs data were available for 1426 women (917 intervention and 509 usual care), based on at least one or more waves of data collection.

Table 1 and Table 2 show mean costs, and mean differences in costs between the intervention and control strategy based on the Perined and questionnaire data.

Table 3 shows the cost-effectiveness results for the composite outcome measure of severe adverse perinatal outcome from a healthcare perspective until one week postpartum (Perined data only).

**Figure 2:**
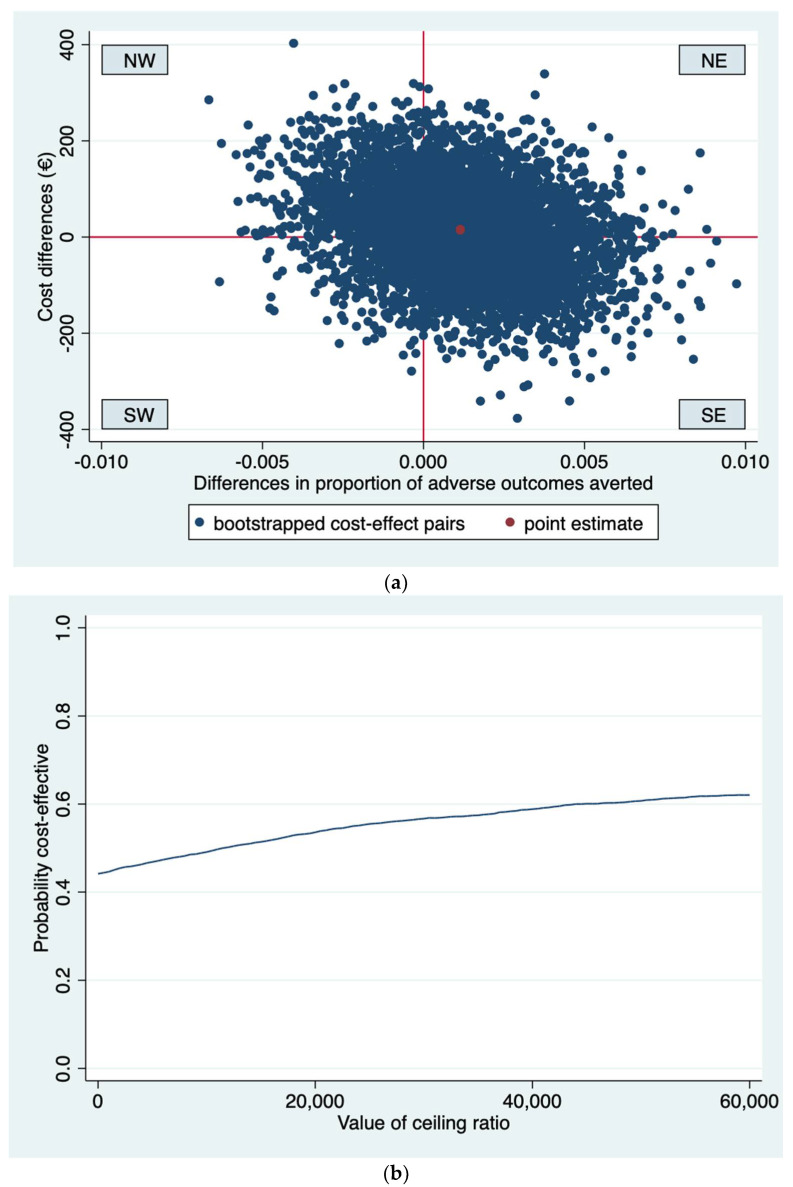
(**a**) Cost-effectiveness plane for analysis 1 (time horizon one week after birth, adverse outcomes averted, Perined data only). The Northeast (NE) quadrant indicates that routine ultrasonography is more effective and more costly than usual care. The Southeast (SE) quadrant indicates that routine ultrasonography is more effective and less costly than usual care. The Southwest (SW) quadrant indicates that routine ultrasonography is less effective and less costly than usual care. The Northwest (NW) quadrant indicates that routine ultrasonography is less effective and more costly than usual care. (**b**) Cost-effectiveness acceptability curve for analysis 1 (time horizon one week after birth, adverse outcomes averted, Perined data only). The y axis shows the probability that routine ultrasonography is cost-effective compared to usual care. The x axis shows the maximum amount of money that society is willing to pay to avert one adverse outcome.

The differences in the prevalence of severe perinatal outcomes and costs between routine third trimester ultrasonography and usual care were not statistically significant. The ICER was 13,404, indicating that to prevent one adverse outcome EUR 13,404 need to be invested in the routine ultrasonography group compared to usual care. The bootstrapped cost-effect pairs were located in all four quadrants of the CE plane, although the majority (69%) were situated in the eastern quadrants of the CE plane (Figure 2a). The CEA curve shows that if society is not willing to invest in the prevention of an adverse outcome, that is, at a ceiling ratio of EUR 0 per adverse outcome prevented, the probability of routine sonography being cost-effective is 0.44 (Figure 2b). This probability increased to 0.54 and 0.57 at ceiling ratios of EUR 20,000 and EUR 30,000 per adverse outcome prevented, respectively.

Table 4 shows pregnancy-related healthcare costs and societal costs for the period from 22 weeks’ gestation until 6 months postpartum for each of the eight pathways, as described in Figure 1. Table 3 also shows the results of the cost-effectiveness analyses based on the extrapolated analyses for the composite outcome measure of severe adverse perinatal outcome from a societal perspective with a time horizon until one week after birth and six months postpartum, respectively. There were no statistically significant differences in costs, adverse outcomes prevented or QALYs between routine ultrasonography and usual care.

**Figure 3:**
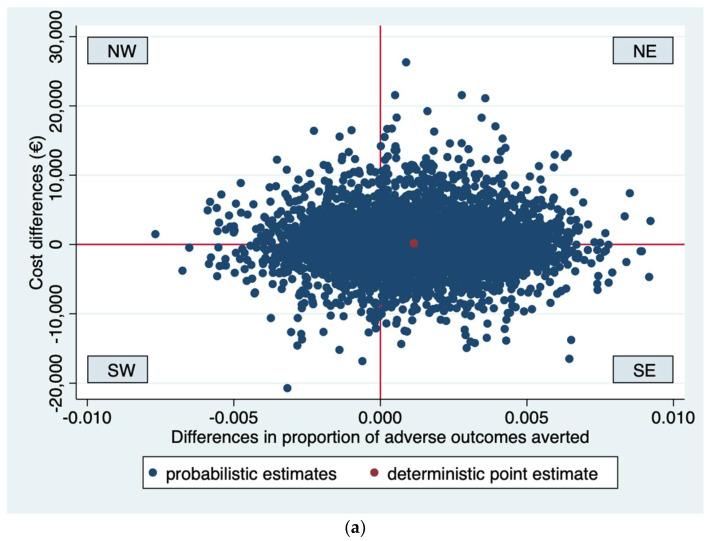

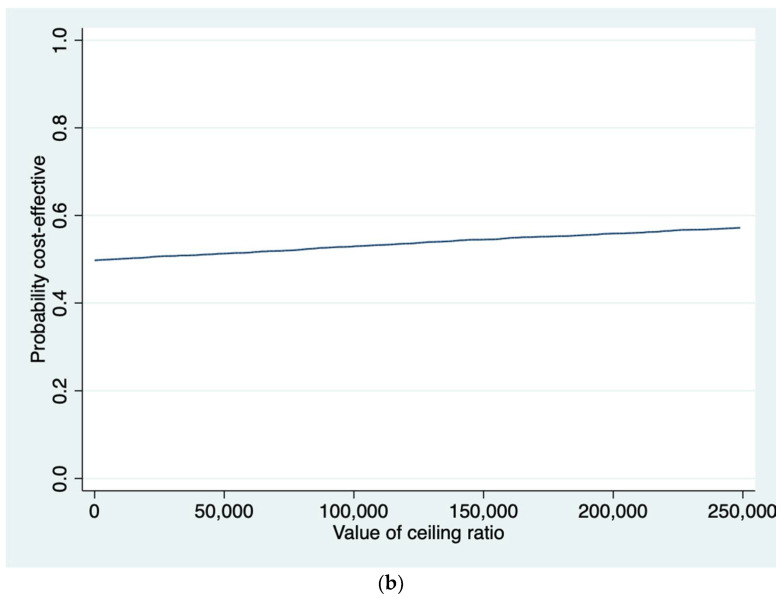
(**a**) Cost-effectiveness plane for analysis 2 (time horizon six months after birth, adverse outcomes averted). The Northeast (NE) quadrant indicates that routine ultrasonography is more effective and more costly than usual care. The Southeast (SE) quadrant indicates that routine ultrasonography is more effective and less costly than usual care. The Southwest (SW) quadrant indicates that routine ultrasonography is less effective and less costly than usual care. The Northwest (NW) quadrant indicates that routine ultrasonography is less effective and more costly than usual care. (**b**) Cost-effectiveness acceptability curve for analysis 2 (time horizon six months after birth, adverse outcomes averted). The y axis shows the probability that routine ultrasonography is cost-effective compared to usual care. The x axis shows the maximum amount of money that society is willing to pay to avert one adverse outcome.

Concerning the extrapolated analysis with a time horizon until 6 months postpartum, the ICER for adverse outcomes prevented was 497,341 indicating that to prevent one adverse outcome EUR 497,341 needs to be invested in routine ultrasonography compared to usual care. As shown in Figure 3a, the bootstrapped cost-effect pairs were located in all four quadrants of the CE plane, although most of the pairs (69%) were situated in the eastern quadrants of the CE plane. The CEA curve (see Figure 3b) showed that the probability that routine ultrasonography is cost-effective compared to usual care is around 0.50 for all ceiling ratios.

For QALYs, the ICER was −8924, indicating that routine ultrasonography is dominated by usual care (i.e., less effective and more costly). The CEA curve (figure not shown) shows that at a ceiling ratio of EUR 0 per QALY gained the probability of cost-effectiveness is 0.49, and that this probability decreases to 0.43 at a ceiling ratio of EUR 20,000 per QALY gained. We found that results based on the extrapolated analyses for the composite outcome measure of severe adverse perinatal outcome from a societal perspective, with a time horizon until one week after birth, were comparable to those using the six month time horizon (see Table 3).

Table 3 also presents the results of the cost-effectiveness analysis based on survey participants (*n* = 1426) only. After 6 months, total societal costs in the routine ultrasonography group were EUR 2497 (95% CI 838; 4204) higher than in the usual care group. However, differences in adverse outcomes and QALYs between groups were small and not statistically significant. The ICER for adverse-outcomes-prevented indicates that on average, EUR 2,236,887 needs to be invested to prevent one adverse outcome in the routine ultrasonography group compared to the usual care group. The CEA curve shows that at a ceiling ratio of EUR 0 per adverse outcome prevented, the probability of routine ultrasonography being cost-effective compared to usual care is 0, and this probability only very slowly increases to 0.10 at a ceiling ratio of EUR 180,000 per adverse outcome prevented (figure not shown). For QALYs, the ICER was −288,558, indicating that routine ultrasonography is dominated by usual care. The probability of cost-effectiveness is 0 at all ceiling ratios for QALYs (figure not shown).

## 4. Discussion

This nationwide cluster-randomized stepped-wedge trial evaluated the cost-effectiveness of third trimester routine ultrasonography compared to usual care from both a healthcare and societal perspective until 6 months postpartum. Routine third trimester ultrasonography combined with usual care did not reduce severe adverse perinatal outcome compared to usual care alone. Costs based on the Perined data and hospital records did not differ between the strategies, while societal costs in the non-selective subsample were significantly higher in the intervention group compared to usual care. There were no statistically significant differences in QALYs between the two strategies either. Cost-effectiveness acceptability curves show that the probability of cost-effectiveness is never higher than 0.6 for all possible ceiling ratios. These results show that third trimester routine ultrasonography on top of usual care was not cost-effective compared to usual care alone.

To the best of our knowledge, only three previous studies investigated the cost-effectiveness of routine ultrasonography during pregnancy and they showed mixed results [32,33]. The Helsinki ultrasound trial (*n* = 9310) showed that one-stage second-trimester ultrasound screening was cost-effective in reducing perinatal mortality as compared to usual care [32]. However, in many Western countries, including the Netherlands, there is no doubt about the use of second trimester ultrasound screening that generally serves a different purpose, i.e., detecting congenital anomalies, as compared to routine third trimester ultrasonography, which is mainly used to monitor fetal growth. Moreover, our study included singleton low-risk pregnant women after second-trimester ultrasound screening. This makes it rather difficult to compare the findings of the Helsinki trial to ours. Wastlund et al., (2019) demonstrated that routine third trimester ultrasonography at approximately 36 weeks’ gestation for the detection and management of macrosomia was not cost-effective as compared to selective ultrasonography, i.e., serial fundal height measurements combined with ultrasonography if clinically indicated, using a decision tree analysis [33]. They estimated probabilities, costs, and health outcomes based on the literature, whereas we used prospectively collected healthcare and societal cost data [33]. A similar decision tree analysis conducted by Wilson et al., (2021) showed that a fetal-presentation only scan (to detect breech) in late pregnancy was cost-effective, while routine ultrasonography assessing fetal size was not [11]. Our study is in line with these previous studies [11,33], and showed that routine third trimester ultrasonography is not cost-effective as compared to usual care, i.e., selective ultrasonography. Thus, based on our results, routine ultrasonography cannot be recommended as a clinical strategy to reduce severe adverse perinatal outcomes. Future trials should address the (cost-)effectiveness of alternative third trimester screening strategies to detect FGR. A promising alternative screening approach includes the use of maternal and placental biomarkers (combined with routine third trimester ultrasonography). These markers have been shown to be associated with adverse perinatal outcomes [34].

Our study has several strengths. The current study is the first multi-center large-scale pragmatic stepped-wedge cluster-randomized trial conducted on a national scale on the cost-effectiveness of routine third trimester ultrasonography in addition to usual care to reduce severe adverse perinatal outcomes in low-risk pregnancies in comparison with usual care alone. The cluster-randomized design reduced the risk of contamination between the treatment strategies, which might introduce bias in individually randomized controlled trials. The stepped-wedge design of the trial reduced confounding resulting from differences between the participating midwifery practices as each practice implemented both treatment strategies for a certain time.

Apart from these strengths, our study also has several limitations. The current study did not achieve the predefined sample size of 15,000 women required to observe statistically significant differences in severe adverse perinatal outcomes between the two study strategies [12]. As a consequence, we were unable to clearly assess whether routine ultrasonography exerts beneficial or detrimental effects on severe adverse perinatal outcomes in comparison with usual care. However, differences between groups were so small that we consider it unlikely that routine ultrasonography may exert such effects on these outcomes as compared to usual care. In addition, although healthcare costs data derived from the Netherlands Perinatal Registry were available for the complete study sample, additional (healthcare) costs data based on hospital records and self-reported societal costs data were available from subsamples only. To estimate additional healthcare and societal costs for the full sample, we conducted a probabilistic sensitivity analysis in combination with Monte Carlo simulation. Moreover, the current study was performed in a country, i.e., the Netherlands, where the primary antepartum care of low-risk pregnancies is provided by highly trained midwives who are officially registered as independent health professionals [35]. Once risk factors are detected or complications occur, midwives refer women to obstetrician-led care. Around 90% of pregnant women in the Netherlands receive midwife-led care in the beginning of pregnancy, and approximately 50% begin labor in midwife-led care [36]. Additionally, the majority of the recommendations incorporated into the multidisciplinary protocol for detecting and managing a suspicion of FGR used in the present study are similar to the recommendations of international guidelines used in other countries, such as the guideline of the Royal College of Obstetricians and Gynaecologist [37]. Therefore, we think that our findings are generalizable to low-risk populations in care settings in other countries, also when the role of midwives is less prominent.

## 5. Conclusions

The current large-scale nationwide stepped-wedge cluster randomized trial with a multidisciplinary protocol for the detection and management of FGR showed that introducing routine third trimester ultrasonography to usual care is not cost-effective in reducing severe adverse perinatal outcomes in low-risk pregnancies compared to usual care alone.

## Figures and Tables

**Table 1:** Mean costs and cost differences in the Perined sample and ultrasounds.

Cost Category	Intervention (*n* = 7026)	Control (*n* = 5948)	Difference (95% CI)
Ultrasound costs ^1^, EUR	72 (0.31)	31 (0.44)	41 (40; 42)
Admission costs child, EUR	966 (66)	1025 (68)	−59 (−252; 126)
Birth costs, EUR	2243 (12)	2211 (13)	32 (−3; 67)
Total Perined costs ^1^, EUR	3584 (68)	3568 (72)	15 (−175; 211)

^1^ Data from the sonographic database were (also) included.

**Table 2:** Pooled mean costs and cost differences in the non-selective questionnaire sample.

Cost Category	Intervention (*n* = 917)	Control (*n* = 509)	Difference (95% CI)
Healthcare costs, EUR	3919 (487)	2757 (163)	1162 (419; 2396)
Lost productivity costs, EUR	7838 (504)	6503 (580)	1335 (−96; 2679)
Total societal costs, EUR	11,757 (753)	9260 (617)	2497 (838; 4204)

**Table 3:** Incremental cost-effectiveness outcomes of routine third trimester ultrasonography with usual care compared to usual care only.

*Type of Analysis*	N	ΔC (95% CI)	ΔE (95% CI)	ICER	CE Plane			
	(Intervention/Control)				NE	SE	SW	NW
*Perined data only, one week after birth, healthcare perspective (Analysis 1)*								
Adverse outcomes prevented	7026/5948	15 (−175; 211)	0.0011 (−0.0033; 0.0058)	13,404	35%	34%	10%	21%
*Extrapolated data, one week after birth, societal perspective (Analysis 2)*								
Adverse outcomes prevented	7026/5948	166 (−7695; 8806)	0.0011 (−0.0034; 0.0057)	145,338	34%	35%	15%	16%
*Extrapolated data, 6 months after birth, societal perspective (Analysis 2)*								
Adverse outcomes prevented	7026/5948	568 (−27,413; 29,419)	0.0011 (−0.0034; 0.0057)	497,341	35%	34%	15%	16%
QALYs	7026/5948	568 (−27,413; 29,419)	−0.064 (−0.24; 0.11)	−8924	14%	12%	37%	37%
*Questionnaire data only, 6 months after birth, societal perspective (Analysis 3)*								
Adverse outcomes prevented	917/509	2497 (838; 4204)	0.0011 (−0.017; 0.019)	2,236,887	54%	0%	0%	46%
QALYs	917/509	2497 (838; 4204)	−0.0087 (−0.019; 0.0015)	−288,558	5%	0%	0%	95%

ΔC = difference in costs (expressed in EUR); ΔE = difference in effects; 95% CI = 95% confidence interval; ICER = Incremental Cost-Effectiveness Ratio; NE = northeast; SE = southeast; SW = southwest; NW = northwest.

**Table 4:** Costs per outcome strategy based on Perined data, hospital records and survey data.

	Perined Costs		Costs Based on Hospital Records				Questionnaire Costs			
			1 Week		6 Months		6 Weeks		6 Months	
	N	Mean (SD)	N	Mean (SD)	N	Mean (SD)	N	Mean (SD)	N	Mean (SD)
Pathway 1	114	16,272 (24,980)	103	1889 (3225)	103	14,548 (16,641)	17	510 (206)	21	13,116 (19,050)
Pathway 2	2775	4797 (6688)	633	1031 (1947)	633	6524 (6314)	309	478 (205)	356	8303 (13,168)
Pathway 3	2	3150 (867)	NA	NA	NA	NA	NA	NA	NA	NA
Pathway 4	4135	2419 (1181)	19	449 (688)	19	2339 (2750)	321	494 (220)	381	6261 (11,486)
Pathway 5	101	13,597 (25,973)	99	1185 (1279)	99	10,097 (9109)	7	427 (120)	7	6447 (13,708)
Pathway 6	2204	5144 (6298)	617	904 (1531)	617	6146 (6101)	150	475 (305)	128	8597 (15,255)
Pathway 7	4	5106 (1815)	4	788 (146)	4	4003 (1533)	1	564 (NA)	1	631 (NA)
Pathway 8	3639	2334 (1193)	40	327 (386)	40	2200 (1928)	199	491 (230)	165	6081 (9914)

## Data Availability

The data presented in this study are available on request from the corresponding author. The data are not publicly available due to the ethical approval provided for the IRIS study, which does not allow publication of individual participant level data.

## Appendix A

The IRIS Study Group: Anneloes L. van Baar, Utrecht University, Utrecht, The Netherlands; Joke M.J. Bais, Medical Center Alkmaar (MCA), Alkmaar, The Netherlands; Gouke J. Bonsel, University Medical Center Utrecht, Utrecht, The Netherlands; Jeroen van Dillen, Radboud University Medical Center, Nijmegen, The Netherlands; Noortje T.L. van Duijnhoven, Radboud University Medical Center, Nijmegen, The Netherlands; William A. Grobman, MD/MBA, Northwestern University, Chicago, USA; Henk Groen, University of Groningen, Groningen, The Netherlands; Chantal W.P.M. Hukkelhoven, Wageningen University & Research, Wageningen, The Netherlands; Trudy Klomp, Amsterdam UMC/AVAG, Amsterdam, The Netherlands; Marjolein Kok, Amsterdam University Medical Center, Amsterdam, The Netherlands; Marlou L. de Kroon, MD PhD, University of Groningen, Groningen, The Netherlands; Maya Kruijt, Dutch Society of Obstetrics and Gynecology NVOG, Utrecht, The Netherlands; Anneke Kwee, University Medical Center Utrecht, Utrecht, The Netherlands; Sabina Ledda, Midwifery practice het Palet/BEN, Rotterdam, The Netherlands; Harry N. Lafeber, Amsterdam University Medical Center, The Netherlands; Jan M.M. van Lith, Leiden University Medical Center, Leiden, The Netherlands; Ben Willem Mol, University of Adelaide, Adelaide, Australia; Bert Molewijk, Amsterdam University Medical Center, Amsterdam, The Netherlands; Marianne Nieuwenhuijze, Academie Verloskunde Maastricht (AVM), Maastricht, The Netherlands; Guid Oei, Maxima Medical Center, Eindhoven, The Netherlands; Cees Oudejans, Amsterdam University Medical Center, Amsterdam, The Netherlands; K. Marieke Paarlberg, Gelre Hospitals, location Apeldoorn, The Netherlands; Eva Pajkrt, Amsterdam UMC, Amsterdam, The Netherlands; Aris T. Papageorghiou, University of Oxford, UK; Uma M. Reddy, Yale University, New Haven, USA; Paul De Reu, Prenataal Screenigscentrum ‘de Meierij’, Eindhoven, The Netherlands; Eva Paijktr, Amsterdam University Medical Center, The Netherlands; Marlies Rijnders, TNO, Leiden, The Netherlands; Alieke de Roon-Immerzeel, Royal Dutch Organization of Midwives (KNOV), The Netherlands; Connie Scheele, University Medical Center Utrecht (UMCU)/BEN, Utrecht, The Netherlands; Sicco A. Scherjon, University Medical Center Groningen (UMCG), Groningen, The Netherlands; Rosalinde Snijders, Amsterdam University Medical Center, Amsterdam, The Netherlands; Marc E. Spaanderman, University Medical Center Maastricht, Maastricht, The Netherlands; Pim W. Teunissen, Maastricht University, The Netherlands; Hanneke W. Torij, Rotterdam University of Applied Sciences, Rotterdam, The Netherlands; Laura Viester, Knowledge Institute of Medical Specialists, Utrecht, The Netherlands; Tanja G Vrijkotte, Amsterdam University Medical Center, Amsterdam, The Netherlands; Janneke Wilschut, Dutch Institute of Clinical Auditing, Leiden, The Netherlands; C. Zeeman, University of Amsterdam, Amsterdam, The Netherlands; Jun Jim Zhang, Shanghai Jiao Tong University School of Medicine, Shanghai, China.

## Appendix B

**Table A1:** Cost categories and unit costs (EUR, 2015) used in the economic evaluation of the IRIS study.

Category	Unit Cost (EUR, 2015)	Source
*Direct healthcare costs*		
Midwife, visit ^1^	33.19	Dutch Costing Guideline 2014, Hakkaart-van Roijen et al., (2015) [18]
General practitioner, visit ^1^	33.19	Dutch Costing Guideline 2014, Hakkaart-van Roijen et al., (2015) [18]
Obstetrician, consultation/Outpatient care	91.55	Dutch Costing Guideline 2014, Hakkaart-van Roijen et al., (2015) [18]
Postpartum outpatient care ^2^	220	https://www.opendisdata.nl/, Netherlands Care Authority (NZA) [23]
Pediatrician/Neonatologist, consultation	101.61	Dutch Costing Guideline 2014, Hakkaart-van Roijen et al., (2015) [18]
Physiotherapist, visit ^1^	33.19	Dutch Costing Guideline 2014, Hakkaart-van Roijen et al., (2015) [18]
Psychologist, visit ^1^	64.39	Dutch Costing Guideline 2014, Hakkaart-van Roijen et al., (2015) [18]
Social worker, visit ^1^	64.39	Dutch Costing Guideline 2014, Hakkaart-van Roijen et al., (2015) [18]
Dietician, visit ^1^	33.19	Dutch Costing Guideline 2014, Hakkaart-van Roijen et al., (2015) [18]
Acupuncturist/Reiki therapist, session ^1^	64.39	Dutch Costing Guideline 2014, Hakkaart-van Roijen et al., (2015) [18]
Other therapist ^1^	33.19	Dutch Costing Guideline 2014, Hakkaart-van Roijen et al., (2015) [18]
Doula session ^1^	54.08	http://degeboortereis.nl/index.php/tarieven/
doula trajectory ^1^	688.30	https://www.oeiikgroei.nl/experts/doula/kosten-en-vergoeding/
Ambulance (for mother or neonate)	518.11	Dutch Costing Guideline 2014, Hakkaart-van Roijen et al., (2015) [18]
Hospital admission, per day		
Hospital admission child, standard care	630.78	Dutch Costing Guideline 2014, Hakkaart-van Roijen et al., (2015) [18]
Hospital admission child, high care	1523.10	Liem, van Baaren, Dellemare et al., (2014) [20]
Hospital admission child, intensive care	1577.50	Liem, van Baaren, Dellemare et al., (2014) [27]
Hospital admission mother, standard care ^2^	478.87	Dutch Costing Guideline 2014, Hakkaart-van Roijen et al., (2015) [18]
Hospital admission mother, medium tertiary care ^2^	645.88	Dutch Costing Guideline 2014, Hakkaart-van Roijen et al., (2015) [18]
Hospital admission mother, intensive care ^2^	1814.89	Liem, van Baaren, Dellemare et al., (2014) [27]
Mode and place of birth		
Home birth	604.00	Royal Dutch Organization of Midwives (2015) [22]
Spontaneous vaginal birth in hospital	2215.00	https://www.opendisdata.nl/ (NZA) [23]
Assisted vaginal birth	2640.00	https://www.opendisdata.nl/ (NZA) [23]
Cesarean section	4210.00	https://www.opendisdata.nl/ (NZA) [23]
Episiotomy	160.03	Vijgen et al., (2011) [26]
Rupture	400.03	Hendrix et al., (2009) [28]
Total rupture	618.26	NZA (2015) [19]
CTG ^2^	45.99	NZA (2015) [19]
Analgesia		
Morphine	0.60	https://www.medicijnkosten.nl/ (National Health Care Institute (ZI)) [21]
Pethidine	0.62	https://www.medicijnkosten.nl/ (ZI) [21]
Benzodiazepine	0.66	https://www.medicijnkosten.nl/ (ZI) [21]
Epidural	187.93	van Baaren, Jozwiak, Opmeer et al., (2013) [27]
General anesthesia	391	NZA (2015) [19]
Blood transfusion ^2^	217.30	Dutch Costing Guideline 2014, Hakkaart-van Roijen et al., (2015) [18]
NIPT ^2^	822.75	https://zorgproducten.nza.nl/ (NZA)
Invasive prenatal diagnostic test ^2^	473,48	https://www.opendisdata.nl/ (NZA) [23]
Advanced ultrasound ^2^	80,48	Dutch Costing Guideline 2014, Hakkaart-van Roijen et al., (2015) [18]
Regular ultrasound	35,03	NZA (2015) [19]
CT-scan ^2^	138.83	Dutch Costing Guideline 2014, Hakkaart-van Roijen et al., (2015) [18]
MRI scan ^2^	217.97	Dutch Costing Guideline 2014, Hakkaart-van Roijen et al., (2015) [18]
Urine screening test ^2^	3.11	NZA (2015) [19]
Urine culture ^2^	29.60	NZA (2014) [19]
Blood culture ^2^	15.00	NZA (2015) [19]
Blood screening test ^2^	3.65	NZA (2015) [19]
Oral Antihypertensive ^2^	7.64	Vijgen et al., (2010) [25]
Contraction inhibitor ^2^	66.01	www.farmacotherapeutischkompas.nl
Pre-/Peripartum antibiotics ^2^	33.07	Vijgen et al., (2011) [26]
Corticosteroids (fetal lung maturation) ^2^	4.99	www.farmacotherapeutischkompas.nl
Fetal neuroprotection (MgSO_4_) ^2^	3.35	www.farmacotherapeutischkompas.nl
Balloon Induction ^2^	177.06	Freeman et al., (2018) [24]
Prostaglandin vaginal gel induction ^2^	18.89	www.farmacotherapeutischkompas.nl
Oxytocin during labor ^2^	0.60	Freeman et al., (2018) [19]
Anti-D Medication during partus ^2^	52.11	NZA (2015) [19]
Neonatal (heart/lung) surgery/complex treatment ^2^	9969	https://www.opendisdata.nl/ (NZA) [23]
Postmortem obduction neonate ^2^	387.05	NZA (2015) [19]
Postmortem blood culture ^2^	29.26	NZA (2015) [19]
Postmortem genetic testing ^2^	869.42	NZA (2015) [19]
Postmortem X-ray ^2^	90.97	NZA (2015) [19]
Long-term observation neonatology ^2^	290	NZA (2015) [19]

Cost categories without symbol indicate cost data derived from the Perinatal Registry of the Netherlands (Perined). ^1^ indicates cost categories derived from the questionnaire study. ^2^ indicates cost categories derived from hospital records.
